# Pediatric Supracondylar Humerus Fractures: Treatment by a Pediatric Orthopedic Surgeon Versus a Non-pediatric Orthopedic Surgeon

**DOI:** 10.7759/cureus.63476

**Published:** 2024-06-29

**Authors:** Joshua Chen, Ally Yang, Melanie Patterson, Ellius Kwok, Gary Updegrove, William Hennrikus

**Affiliations:** 1 Orthopedics, Penn State Health Milton S. Hershey Medical Center, Hershey, USA; 2 Orthopedics, Yale School of Medicine, New Haven, USA; 3 Orthopedics, Prisma Health Greenville Memorial Hospital, Greeneville, USA; 4 General Surgery, Thomas Jefferson University Hospital, Philadelphia, USA

**Keywords:** outcomes, fracture, supracondylar, pediatric, elbow

## Abstract

Introduction: Supracondylar humerus fractures are the most common surgically treated fracture in children. National trends have demonstrated an increase in transfers of supracondylar fractures to pediatric hospitals due to the perception that supracondylar fractures need to be treated by pediatric specialists. The objectives of the study are to compare the outcomes of surgically treated pediatric supracondylar humerus fractures (PSCHF) between a pediatric orthopedic surgeon and a non-pediatric orthopedic surgeon at a single academic medical center; to assess radiographic reduction, the number of pins used, surgical time, Flynn criteria outcomes, and complications associated with PSCHF treatment by both types of surgeons; to determine if there is a significant difference in outcomes between pediatric and non-pediatric orthopedic surgeons in the treatment of PSCHF.

Methods: Forty-seven consecutive pediatric patients, with an average age of 5.5 years old, who had undergone surgical correction for supracondylar humerus fractures during 2019 were included in this study. The intervention performed was closed reduction and percutaneous pinning. The main outcome measured: radiographic reduction on the AP and lateral X-ray view, number of K wires used, use of a medial K wire, time of surgery, the Flynn criteria, and complications. The Human Research Protection Program (HRPP) at Penn State approval was obtained.

Results: Radiographic reductions as measured by Bauman’s angle and the position of the anterior humeral line were excellent and similar between surgeons. The pediatric orthopedic surgeon used more medial K wires (p=0.0007), fewer K wires (p=0.0065), and the length of surgery was shorter (p=0.019). The Flynn criteria were similar with equal excellent and good results. For both surgeons, no complications such as loss of reduction, infection, iatrogenic nerve injury, compartment syndrome, or cubitus varus occurred.

Conclusions: Outcomes of treatment of displaced PSCHF by the pediatric and non-pediatric orthopedic surgeons were equal. The results of this study reaffirm the assertion that both pediatric and non-pediatric orthopedic surgeons have sufficient training and skill to treat these common fractures, thereby contributing to a more informed decision-making process in clinical practice.

## Introduction

Pediatric supracondylar humerus fractures (PSCHF) are the most common elbow fracture and the most common surgically treated fracture in children [1,2]. PSCHF typically occurs when a healthy six-year-old sustains a simple fall [3]. Diagnosis and treatment of these fractures is a core competency milestone per the Accreditation Council of Graduate Medical Education (ACGME). Every orthopedic surgery resident must demonstrate competency in the treatment of a supracondylar fracture before graduating [4]. For example, per the ACGME, the graduating orthopedic resident must understand the indications for closed and open reduction, understand how to avoid complications, be capable of performing closed reduction or open reduction and pinning, and be capable of treating complications such as wound problems, infections, and compartment syndrome [4].

Non-displaced PSCHFs can be treated with a cast or splint [5]. Displaced PSCHFs are usually treated with closed reduction and percutaneous pinning or open reduction and pinning [2]. National trends have demonstrated an increase in transfers of this fracture to pediatric hospitals due, in part, to the perception that PSCHF must be treated by a pediatric orthopedic surgeon [6-9]. The purpose of this study is to compare the outcomes of surgically treated PSCHF between a pediatric orthopedic surgeon and a non-pediatric orthopedic surgeon at a single center, to assess radiographic reduction, the number of pins used, surgical time, Flynn criteria outcomes, and complications associated with PSCHF treatment by both types of surgeons, and to determine if there is a significant difference in outcomes between pediatric and non-pediatric orthopedic surgeons in the treatment of PSCHF.

## Materials and methods

The study was approved by the Human Research Protection Program (HRPP) at Penn State. Informed consent and confidentiality were performed for each surgery as required by the medical center. Forty-seven displaced PSCHF treated by two orthopedic surgeons at the same hospital in 2019 were retrospectively reviewed. At our pediatric referral hospital, PSCHF is treated by pediatric and non-pediatric orthopedic surgeons. Inclusion criteria included any pediatric patients (<12 years of age) with a surgically treated supracondylar fracture treated by the two attending orthopedic surgeons during the year 2019. Exclusion criteria included non-displaced fractures and patients >12 years of age. Fractures were radiographically graded by the modified Gartland classification [10]. Data was collected by fourth-year medical students and included age, gender, nerve palsies, presence of a pulse, pucker sign, any additional fractures, open reductions, number of pins, quality of reduction, average length of surgery, length of hospital stay, time to final follow up, and complications. Follow-up by both orthopedic surgeons was performed at one week, one month, and two months post operation with repeat radiographs at each appointment. The quality of reduction was graded by two radiographic measurements and reviewed by the attending orthopedic surgeon. On the lateral view, did the anterior humeral line intersect the capitellum [11], and on the AP view, was Baumann’s angle between 64 and 78 degrees? Baumann’s angle was defined as the angle between the axis of the humeral shaft and a line parallel to the physis of the lateral condyle [12].

An A grade was given if, on the lateral radiograph, the anterior humeral line intersected the capitellum. If not, the grade was B. A second A grade was given if, on the AP radiograph, the Baumann’s angle was equal to 64-78 degrees. If not, the grade was B. Clinical outcomes were graded by the Flynn criteria measuring the carrying angle and the elbow range of motion [13]. The carrying angle was defined as the angle between the axis of the humeral shaft and the axis of the forearm with the elbow in full extension, the forearm in supination, and the patient supine [14].

Surgical technique

The surgical technique was similar for both the pediatric and non-pediatric orthopedic surgeons with the exception of placing a medial pin. The patient was under general anesthesia and positioned supine on the operating room table with the affected arm on a radiolucent arm board. The fluoroscopy C-arm was placed under the arm board. Reduction and lateral pinning were performed as previously described by Smuin et al. [15]. The fracture was reduced by traction, translation, and flexion. Lateral pins were then placed under fluoroscopic guidance.

When a medial K wire was determined clinically indicated by the pediatric orthopedic surgeon the medial K wire was placed per the technique previously described by Edmonds et al. [16]. The lateral K wire was placed first with the elbow in flexion. Then, with the elbow extended to less than 90 degrees of flexion, the surgeon thumb palpated the ulnar nerve and pushed it posteriorly behind the medial epicondyle to prevent iatrogenic ulnar nerve injury. The K wire was then introduced via the medial epicondyle. 

Statistics and analysis 

Comparative analysis between the pediatric and non-pediatric orthopedic surgeon groups was made using Microsoft Excel 2021. Two unpaired t-tests assuming equal variances were run comparing the pediatric and non-pediatric orthopedic surgeons; the first compared the number of pins used and the second compared the time length of surgery in minutes. In addition, a chi-square test was performed comparing the use of medial and lateral pins versus lateral pins only between the pediatric and non-pediatric trained orthopedic surgeons. A p-value less than 0.05 was considered statistically significant.

## Results

The outcomes of cases treated by two surgeons were compared. Thirty-two of the 47 patients (68%) had been transferred from another hospital for surgical care. The average time from receiving the transfer request call to the patient arriving at the pediatric hospital was 2 hours and 45 minutes (range 1 hour 50 minutes to 5 hours 10 minutes). No complications occurred due to the delay in care.

**Figure 1:**
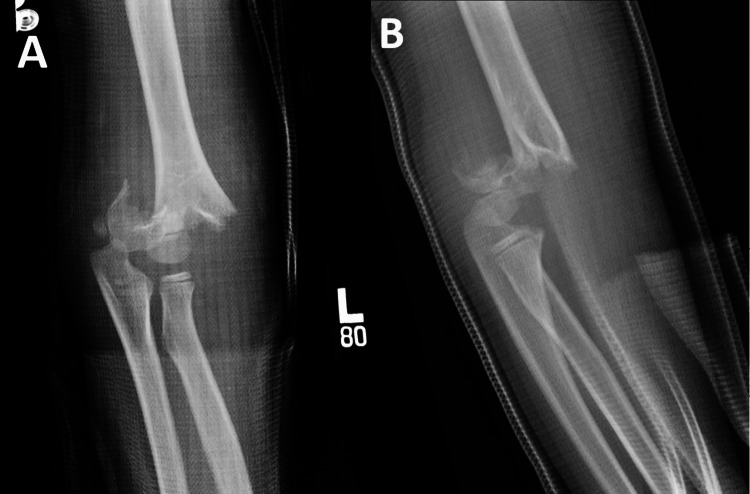
Example case performed by a pediatric surgeon. Injury X-rays. Injury X-rays of an example Gartland type III treated by the pediatric orthopedic surgeon A) AP and B) lateral.

**Figure 2:**
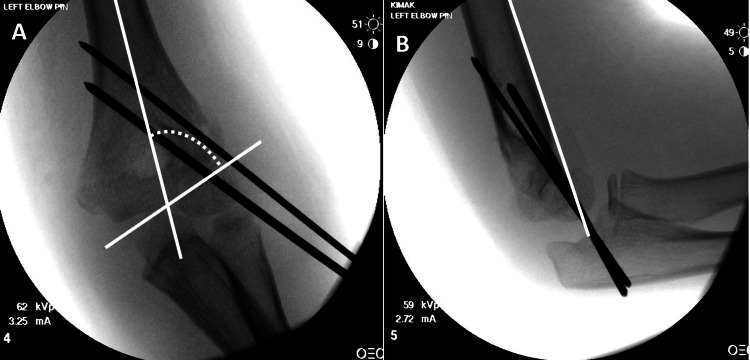
Example case performed by the pediatric orthopedic surgeon. Post-operative X-rays. Example post-operative X-rays from the pediatric orthopedic surgeon A) AP X-ray showing measurement of Baumann’s angle of 70 degrees B) lateral X-ray demonstrating the anterior humeral line intersecting the capitellum.

**Figure 3:**
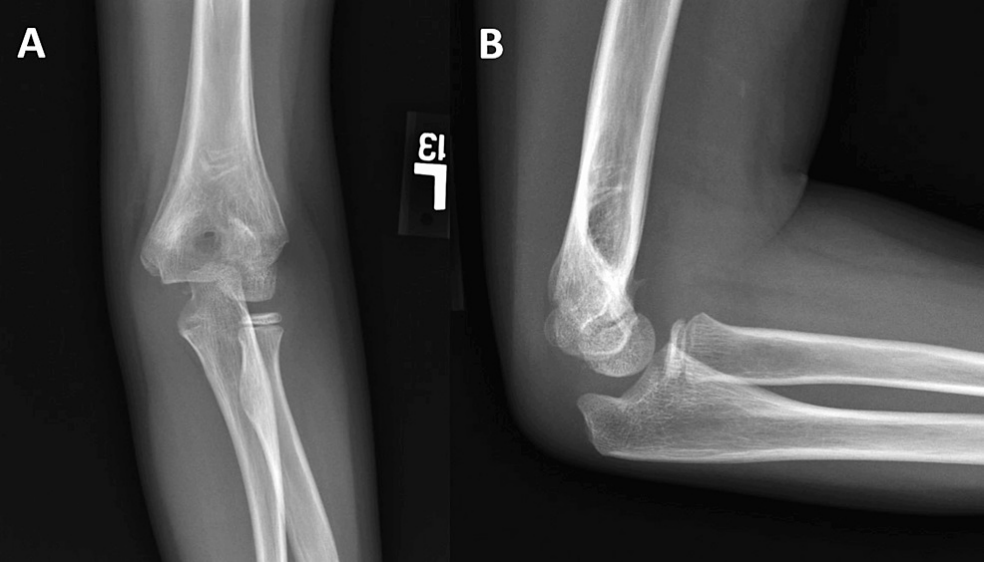
Example case performed by the pediatric orthopedic surgeon. Final follow-up X-rays. Example case performed by the pediatric orthopedic surgeon of final follow-up A) AP and B) lateral elbow X-rays at three months post operation, showing well-healed supracondylar fracture.

The pediatric orthopedic surgeon had 30 years of clinical experience and completed a pediatric fellowship. In the current series, this surgeon treated 31 patients: 17 were male and 14 were female. The average patient age was 5.5 years (range: 3-9 years). Twenty-one Gartland type 2 and 10 type 3 fractures were treated. Three patients had an additional distal radius fracture. Three transient nerve palsies, one pulseless limb, and one pucker sign occurred at the time of injury. The average number of pins utilized was 2. Eleven medial pins (35%) were placed per the technique described by Edmonds et al. [16]. The average length of surgery was 28 minutes. The quality of the radiographic reduction demonstrated that in all 31 cases, the anterior humeral line intersected the capitellum (A grade) and the Baumann’s angle was between 64-78 degrees (A grade) (Figures 1, 2, 3). The hospital stays after surgery averaged 17 hours. The average time in a cast was 29 days. The average length of follow-up was 14 weeks (range: 10-21 weeks). By the Flynn criteria, outcomes were 28 excellent and 3 good.

**Figure 4:**
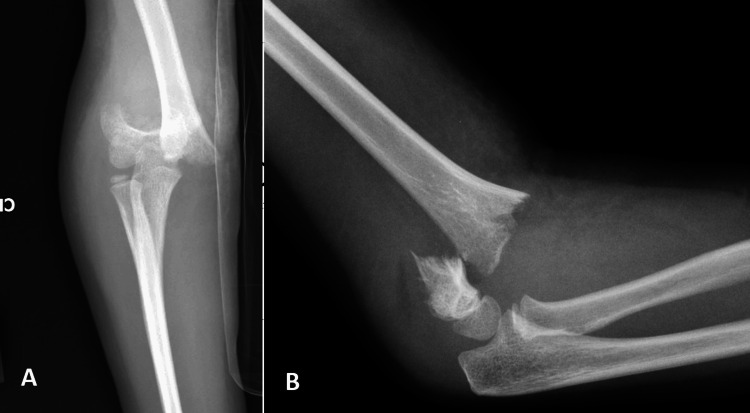
Example case performed by the non-pediatric orthopedic surgeon. Injury X-rays. Injury X-rays of an example Gartland type III treated by the non-pediatric orthopedic surgeon A) AP and B) lateral.

**Figure 5:**
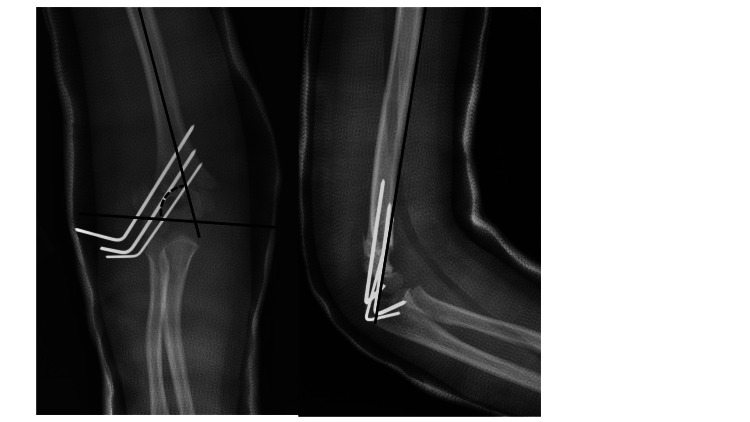
Example case performed by the non-pediatric orthopedic surgeon. Post-operative X-rays. Example post-operative X-rays from the non-pediatric orthopedic surgeon. A) AP X-ray showing measurement of Baumann’s angle of 66 degrees. B) Lateral X-ray demonstrating the anterior humeral line intersecting the capitellum.

**Figure 6:**
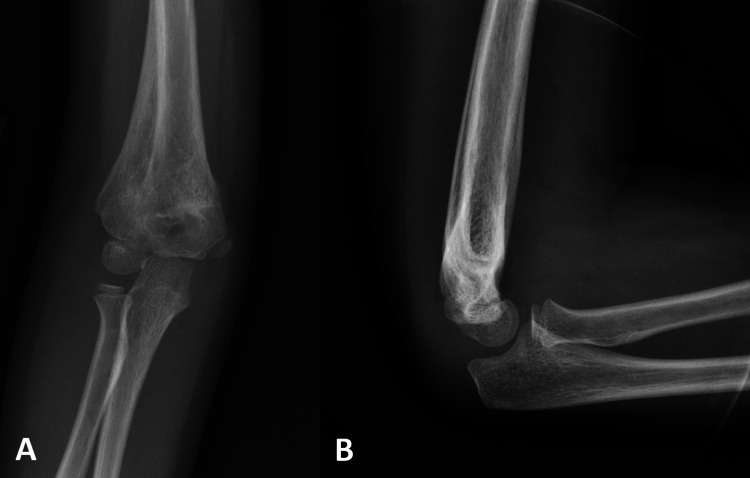
Example case performed by the non-pediatric orthopedic surgeon. Final follow-up X-rays. Example case performed by the non-pediatric orthopedic surgeon of final follow-up A) AP and B) lateral elbow X-rays at three months post operation, showing well-healed supracondylar fracture.

The non-pediatric orthopedic surgeon had one year of clinical experience and had completed an adult trauma fellowship and a shoulder fellowship. In the current series, this surgeon treated 16 patients: 8 were male and 8 were female. The average patient age was 5.8 years (range: 3-9 years). Six Gartland type 2 and 10 type 3 fractures were treated. No associated fractures occurred. Two transient nerve palsies, 1 pulseless limb, and 4 pucker signs occurred at the time of injury. The average number of pins utilized was 2.7. Three lateral pins were utilized in 12/16 (75%) of fractures. No medial pins were placed. The average length of surgery was 37 minutes. The quality of the radiographic reduction demonstrated that in all 16 cases, the anterior humeral line intersected the capitellum (A grade) and the Baumann’s angle was between 64 to 78 degrees (A grade) (Figures 4, 5, 6). The hospital stays after surgery averaged 12 hours. The average time in the cast was 25 days. The average length of follow-up was 10 weeks (range: 8-19). By the Flynn criteria, outcomes were 14 excellent and 2 good.

In the current study, the non-pediatric orthopedic surgeon did not use any medial K wires; the pediatric orthopedic surgeon utilized a medial K wire in 11/31 (35%) cases. No iatrogenic ulnar nerve palsies occurred. In the current study, the non-pediatric orthopedic surgeon used three lateral pins in 12/16 (75%) cases. Overall, in the current study, the pediatric orthopedic surgeon utilized more medial pins (p=0.0065), fewer total pins (p=0.00071) (Table 1), and the length of surgery was 12 minutes faster (p=0.019) (Table 2).

**Table 1:** Data analysis comparing the number of pins between pediatric versus non-pediatric orthopedic surgeon.

	Pediatric fellowship-trained orthopedic surgeon	Non-pediatric fellowship-trained orthopedic surgeon
Mean (number of pins)	1.97	2.75
Standard deviation	0.80	0.45
T statistic	-3.64	
P-value for unpaired t-test	0.00071	

**Table 2:** Data analysis comparing operative time in minutes between pediatric versus non-pediatric fellowship-trained orthopedic surgeon.

	Pediatric fellowship-trained orthopedic surgeon	Non-pediatric fellowship-trained orthopedic surgeon
Mean (minutes)	28.20	36.75
Standard deviation	11.65	11.06
T statistic	-2.43	
P-value for unpaired t-test	0.019	

In the two patients that presented with pulseless limbs (one treated by each surgeon), the pulse returned after closed reduction, pinning, and extension of the elbow. No open reductions were performed by either surgeon. The quality of closed reductions graded by radiographs for all cases, by both surgeons, were "A" for the position of the anterior humeral line and "A" for the Baumann’s angle. No case of infection, compartment syndrome, loss of reduction, post-operative nerve injury, cubitus varus deformity, re-admission, or other complications occurred. The five traumatic nerve palsies resolved spontaneously by 21 weeks (Table 3).

**Table 3:** Summary of data comparing the pediatric versus non-pediatric orthopedic surgeon.

	Pediatric orthopedic surgeon	Non-pediatric orthopedic surgeon
Cases	31	16
Average age	5.5 ± 2.8 y	5.8 ± 2.5 y
Gartland type II	21	6
Gartland type III	10	10
Additional fractures	3 distal radius	None
Nerve palsies	3	2
Pulseless	1	1
Pucker sign	1	4
Duration of surgery	28 ± 12 min	37 ± 11 min
# Pins placed/case	2 ± 0.8	2.7 ± 0.4
# medial pins	11	0
Quality of reduction	A/A	A/A
Hospital stay	17 ± 6.1 hours	12 ± 3.2 hours
Time in cast	29 ± 3.5 days	25 ± 3.0 days
Average follow up	14 ± 8 weeks	10 ± 4 weeks

## Discussion

Controversy exists about whether radiographic outcomes, clinical outcomes, and complications of PSCHF differ in patients treated by a pediatric orthopedic surgeon compared to a non-pediatric orthopedic surgeon [6-9]. Treatment of a pediatric supracondylar fracture is a core ACGME competency milestone that every orthopedic surgery resident must pass before graduating [4]. Despite the common occurrence of PSCHF and the universal training in the care of these injuries by all orthopedic residents, a practice trend has developed over the past two decades in which children with PSCHF are transferred from community hospitals to pediatric hospitals based on the perception that this fracture must be treated by a pediatric orthopedic surgeon [1,3,6,9,17]. For example, in the current study, 68% of PSCHF treated were transferred to our pediatric children’s hospital for surgical care.

Inter-facility transfers cause delays in care [1,7,8]. In 2018, Nielsen et al. reported an average time from transfer acceptance to hospital admission of 3.8 hours (range of 1.1 to 9.7 hours) [18]. Ramachandran et al. suggested that delays can lead to an increase in morbidity in the form of compartment syndromes [19]. In addition, Macnab demonstrated that transfer delays cause inconvenience to families due to increased costs, more complex travel arrangements, and, in some cases, the need for hotel accommodations for the family [20]. On the other hand, treatment at a community hospital without referral to a pediatric center can decrease anxiety, stress, and emergency department waiting time for the patient and his/her family [21]. In the current study, the average delay in care due to the transfer was 2 hours and 45 minutes. No increase in morbidity occurred from delays due to transfers in the current study. Family inconvenience or stress was not studied.

We report no difference in radiologic results, clinical outcomes, or complications in the management of patients with PSCHF when compared to a pediatric and non-pediatric orthopedic surgeon. The quality of reduction was excellent and similar between surgeons comparing the alignment of the anterior humeral line and Baumann’s angle. These findings support previous literature reporting no difference in Baumann’s angle measurements comparing fractures treated by pediatric and non-pediatric orthopedic surgeons [17,22,23]. The complication rates of operative PSCHF are less than 5% with the most common being pin migration, loss of reduction, broken pin, cubitus varus, and superficial infection. The complication rate was minimal in the current study and similar to literature standards [24]. The current study demonstrates equivalent outcomes despite the subspecialty training and experience of the surgeon. These results support the findings of previous authors [1,8,17,22,23]. The perception that PSCHF must be treated by a pediatric orthopedic surgeon is a misperception. The findings of the current study can be useful in areas where pediatric orthopedic surgeons are not available.

Multiple authors have reported that crossed-pin fixation provides a more stable fixation construct than all lateral entry K wire fixation [25,26]. Although, loss of reduction occurs more commonly using only lateral pins [26]. Placing a third lateral K wire can improve stability if a medial K wire is not used [27]. The American Academy of Orthopaedic Surgeons Appropriate Use Criteria recommends all laterally based pin fixation due to the risk of iatrogenic ulnar nerve palsy stemming from placing a K wire through the medial epicondyle [28]. Although a medial K wire is not needed in most cases, surgeons should perform the operation best suited to obtain adequate fracture stability, including the use of a medial K wire if needed [16]. In the current study, the non-pediatric orthopedic surgeon did not use any medial K wires; the pediatric orthopedic surgeon utilized a medial K wire in 11/31 cases (35%) of cases. No iatrogenic ulnar nerve palsies occurred. Although these factors were statistically significant, they were not of any practical significance and did not change outcomes or complications. For both surgeons, no case of loss of reduction, iatrogenic nerve injury, compartment syndrome, or cubitus varus occurred.

Last, value = outcomes/cost [29]. Achieving high value for patients is one of the primary goals of orthopedic health care delivery. Minimizing non-value-added steps in healthcare delivery can reduce costs and potentially improve value [29]. For example, reducing unneeded emergency department transfers can improve value. The estimated average cost of a pediatric emergency department transfer is $4843 [30]. In addition, Shasti et al. reported that operating room, anesthesia, and radiology costs were 44% less (about $2200) when the child’s fracture was treated at a community hospital compared to a children’s hospital [30]. Prior authors have concluded, that when there is a qualified non-pediatric orthopedic surgeon available at the community hospital, the value of orthopedic care for PSCHF can be increased by providing treatment at the community hospital rather than transferring the patient for care to a children’s hospital [8,20,21,31].

The limitations of the current study include the retrospective design, which leads to inherent biases and limitations in data collection. Cases performed at a single hospital may limit the extrapolation of results to other settings. Limited cohort size, which may result in poor generalizability, short follow-up, and cases studied by only two surgeons. A future randomized prospective study is recommended to further elucidate comparisons of treatment outcomes for PSCHF by pediatric and non-pediatric orthopedic surgeons.

## Conclusions

In conclusion, the outcomes of PSCHF treated by a pediatric orthopedic surgeon and a non-pediatric orthopedic surgeon were the same. Pediatric subspecialty training is not a prerequisite for successfully treating PSCHF. The results of this study reaffirm the assertion that both pediatric and non-pediatric orthopedic surgeons have sufficient training and skill to treat this common fracture, thereby contributing to a more informed decision-making process in clinical practice.
